# Multifaceted community health education programs as powerful tools to mitigate snakebite-induced deaths, disabilities, and socioeconomic burden

**DOI:** 10.1016/j.toxcx.2022.100147

**Published:** 2022-12-26

**Authors:** Sakthivel Vaiyapuri, Priyanka Kadam, Gnaneswar Chandrasekharuni, Isadora S. Oliveira, Subramanian Senthilkumaran, Anika Salim, Ketan Patel, Jacqueline de Almeida Gonçalves Sachett, Manuela B. Pucca

**Affiliations:** a School of Pharmacy, University of Reading, Reading, RG6 6UB, UK; b Snakebite Healing & Education Society, Mumbai, India; c Madras Crocodile Bank Trust, Mamallapuram, Tamil Nadu, India; d Department of Biomolecular Sciences, School of Pharmaceutical Sciences of Ribeirão Preto, University of São Paulo, Ribeirão Preto, Brazil; e Manian Medical Centre, Erode, Tamil Nadu, India; f School of Biological Sciences, University of Reading, Reading, RG6 6UB, UK; g Nurse School, State University of Amazonas, Manaus, Amazonas, Brazil; h Medical School, Federal University of Roraima, Boa Vista, Brazil

**Keywords:** Snakebite envenoming, Community health education, Socioeconomic impact, Public awareness, Rural communities, Healthcare professionals, health policies

## Abstract

Snakebite envenoming (SBE) predominantly affects rural impoverished communities that have limited access to immediate healthcare. These communities often hold numerous myths/misbeliefs about snakes and SBE. Moreover, healthcare professionals who practice in rural regions often work in unstable situations with limited medical infrastructure and therefore, lack sufficient knowledge/experience and confidence in the clinical management of SBE. Due to the lack of reliable statistics on the true burden of SBE, developing health policies for this condition by relevant authorities may be difficult. Hence, it is critical to improve awareness about SBE among rural communities, healthcare professionals and health authorities using robust multifaceted community health education approaches. Here, we describe the design, development, implementation, and impact of distinctive community health education approaches that we used in India and Brazil. A wide range of educational tools including information leaflets, posters, pocket guides, learning materials for healthcare professionals and short/long video documentaries were developed in local languages and used to engage with target communities through direct assemblies as well as mass/traditional and social media. Notably, we used diverse methods to determine the impact of our programs in improving awareness, treatment-seeking behaviour, and clinical practice. The people-centred approaches that we used were inclusive and highly impactful in instigating fundamental changes in the management of SBE among rural communities. The resources and approaches presented in this article can be easily adapted for wider use in other countries in order to collectively reduce SBE-induced deaths, disabilities and socioeconomic ramifications.

## Highlights

• Lack of awareness is a key factor in augmenting snakebite (SBE)-induced deaths.
• We report community education programs as a powerful tool to mitigate SBE.
• Diverse tools and people-centred approaches were used to engage communities.
• These were successful in changing the treatment-seeking behaviour of victims.
• The tools and approaches can be adapted for other countries to mitigate SBE.

## Graphical abstract

**Figure:**
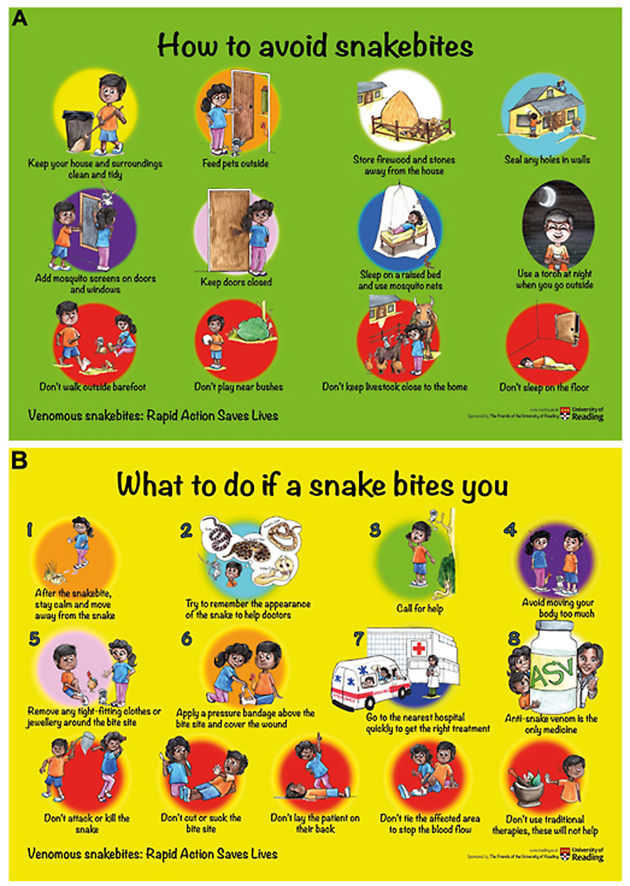

## 1 Introduction

Snakebite envenoming (SBE) is a high-priority neglected tropical disease that predominantly affects rural communities living in low- and middle-income countries in Asia, Latin America, and Africa (Gutiérrez et al., 2017; Williams et al., 2019). SBE causes around 140,000 deaths and 500,000 permanent disabilities annually worldwide (Kasturiratne et al., 2008; Longbottom et al., 2018). Notably, SBE instigates substantial socioeconomic impacts on victims, their families and society through resulting consequences including deaths, permanent disabilities, psychological morbidity, and significant treatment cost (Vaiyapuri et al., 2013; Franco et al., 2022). Many people including children survive SBE but may suffer from permanent disabilities that need long-term or lifelong care. The majority of SBE victims are from underprivileged backgrounds with limited access to advanced healthcare due to geographic and economic circumstances (Harrison et al., 2009; Vaiyapuri et al., 2013; Cristino et al., 2021). Although SBE is a treatable condition, the victims often do not seek prompt treatment in appropriate medical settings due to various factors (Harrison et al., 2009; Williams et al., 2017). For example, most victims may not have access to immediate medical or transport facilities, seek treatments from locally available traditional healers and practice inappropriate first aid measures. These factors exacerbate SBE-induced complications which impact the likely success of any treatment and inflate the treatment cost (Williams et al., 2017). Hence, encouraging, and empowering SBE victims to seek prompt hospital treatment is key to mitigating SBE-induced deaths and disabilities (Samuel et al., 2020). Similarly, improving primary healthcare settings with adequate facilities to handle SBE victims will be an important step in tackling this issue. SBE can also be prevented by taking simple but significant precautionary measures such as using appropriate clothing, footwear and the use of torches when going out in the dark (Kadam et al., 2021).

The World Health Organisation (WHO) has developed a strategic roadmap to reduce SBE-induced deaths and disabilities in half by 2030 (Williams 2019). The WHO's four key pillars of their strategy to decrease the burden of SBE include (1) empowering and engaging the communities, (2) ensuring safe and effective treatment, (3) strengthening health systems, and (4) increasing partnerships, coordination, and resources with strong collaborations. To achieve these goals, robust multifaceted/trans-disciplinary approaches with a broad spectrum of stakeholders are required (Gutiérrez et al., 2022), especially to empower and engage communities with the right information on the prevention and control of the SBE (Samuel et al., 2020; Kadam et al., 2021). Here, we describe the design and development of resources for multifaceted community health education programs and their implementation in India and Brazil. Importantly, we measure their impact by studying behavioural changes among rural populations in these countries. Moreover, we emphasise the need for integrated responses from scientists, research organisations and non-governmental organisations (NGOs) in influencing policies and guidelines for SBE in collaboration with local governments and other relevant authorities.

## 2 The necessity to improve SBE awareness

The epidemiology of SBE is difficult to establish, which makes it a challenging task to control and mitigate incidents in comparison to other common and tropical diseases. SBE mostly affects workers and their families who rely on agriculture-related occupations in rural and tribal areas (Vaiyapuri et al., 2013; Gutiérrez et al., 2017; Babo Martins et al., 2019). The daily activities of rural dwellers expose them to snakes and therefore the likelihood of SBE. Many SBE victims may have little or no access to immediate medical assistance, health care or appropriate educational programs, which often delay the essential treatment and augment SBE-induced complications (Williams et al., 2017). The literacy rate among rural communities may be another limiting factor which warrants additional support as well as access to care closer to their living areas. Moreover, several rural and tribal villages may not have appropriate road or transport facilities, making it difficult for them to access healthcare even if they chose to. The availability of local traditional healers and their familiarity with the communities is likely to be another concern as SBE victims are often encouraged or forced to seek treatments from them instead of modern medical assistance (Nann 2021). While the traditional healers may have experience in treating some ailments including a dry bite from a venomous snake or non-venomous snakebites (Pucca et al., 2020), they often refer the patients or stop treating them when they develop significant envenomation symptoms. Since SBE can induce a range of complications in the body (Warrell 2010), it needs proper care in an appropriate medical setting that contains the required facilities for treatment including ventilation support. Therefore, it is crucial to educate these communities about the importance of snakes to the ecosystem, how to avoid conflicts with snakes and the necessity to seek prompt and suitable medical care for SBE. The use of inclusive and targeted tools specifically designed to meet the needs of rural communities and circumstances is key for engagement and ultimately in realising impact (Samuel et al., 2020). Moreover, community engagement activities require individuals, local government/health authorities or NGOs who are familiar with the communities they serve in order to develop trust in the programme that is being advocated, otherwise, they may not engage or implement changes due to a lack of trust in external people or agencies (Kadam et al., 2021).

The only effective treatment available for SBE are the antivenoms produced against the venom(s) of one or a few medically significant snakes in specific regions/countries. Although antivenoms are known to induce some adverse effects such as anaphylaxis and serum sickness, their administration is critical to save lives (de Silva et al., 2016). Governments in several countries provide antivenoms free of charge. However, the healthcare professionals who practice in rural areas often do not have the confidence in handling SBE victims and administering antivenoms (Williams et al., 2017). Therefore, they often refer the patients to distant tertiary care settings, which may take several hours to reach. This delays the essential treatment and augments the complications. Moreover, the primary healthcare settings in rural regions may not be suitably equipped with all the necessary facilities, discouraging healthcare professionals from treating SBE victims. However, they need to understand that providing essential first aid and at least a few vials of antivenom will significantly improve the survival rate. Therefore, in addition to the vulnerable communities, healthcare professionals need urgent training and support to improve their awareness and skills to confidently provide immediate medical support for SBE victims.

The lack of reliable statistics on country specific SBE incidents is a significant limiting factor in developing appropriate health policies with dedicated funding support. Moreover, there are no specific mechanisms to collect SBE statistics in many countries, particularly where many people seek treatments from private healthcare and locally available traditional healers. Similarly, the availability of healthcare insurance for people in rural areas and the affordability for these people if available, is likely to be another issue. A single but significant medical (e.g., SBE) treatment cost can alter the lives of an individual and their family (Vaiyapuri et al., 2013). Lack of infrastructure, the unavailability of continuing medical education (CME) training for medical staff and appropriate security measures for the medical staff when patients’ condition worsens are likely to be some of the key factors for a high number of referrals to tertiary hospitals and resulting deaths. Moreover, there is a need for local emergency response teams within the communities and local government administration. A robust ambulance service system in remote locations with short response times could be another key element in saving lives of SBE victims. Hence, it is important to raise awareness among government officials including healthcare authorities as well as insurance providers to develop robust policies to improve the healthcare systems and their affordability for SBE victims from all regions.

Due to the nature of community health education programs, the timeline to impact through behavioural and policy changes may be a slow process which might take a few years with the persistent influence of government agencies, rural health workers and members of the communities. However, providing key awareness through multifaceted approaches at different levels is essential to change the treatment-seeking behaviour of rural communities and their lifestyle to prevent SBE incidents (Samuel et al., 2020). Although people may take some time to adapt, they will become accustomed to the new lifestyle and eventually change their behaviour. For example, everyone knows that electricity is dangerous, however, they learned how to live with it. Similarly, community health education programs will empower communities to live with venomous snakes, reducing human-snake conflicts and incidence rates, mitigating the consequences of SBE.

## 3 SBE in India and Brazil

In this article, we mainly focus on the community health education programs that we performed in India and Brazil. Therefore, we outline the context of SBE in India and Brazil in more detail to illustrate its burden and the need for robust community education programs.

India is the largest democratic country in the world with an estimated population of 1.39 billion (Ellis-Petersen, 2022)(India, 2022a). It is comprised of 28 states and 8 union territories (Government of (India, 2022b) ). Each state is subdivided into several administrative districts and further split into taluks that comprise several rural panchayat villages. The majority of people in India may live in rural areas, and they are most likely to be involved in agricultural activities (Government of India, 2022a). Therefore, they may encounter snakes often at the workplace and around their living areas. A previous study demonstrated that small and medium-sized villages with up to 250 houses are likely to encounter snakes more frequently than larger villages and urban areas with more than 250 houses (Vaiyapuri et al., 2013). Generally, India is considered ‘the capital of SBE’ due to the high number of incidents and deaths. India accounts for around 58,000 SBE-induced deaths every year (Suraweera et al., 2020; Roberts et al., 2022; Livemint, 2022) although the actual number may undoubtedly be much higher as many victims do not seek hospital treatment or die on the way to hospitals (Vaiyapuri et al., 2013). Such deaths are likely to be unaccounted for as there are no available records in hospitals, local police stations or traditional treatment centres (Kadam et al., 2021). Moreover, a large proportion of SBE victims may seek treatment in private healthcare settings, where the incidence rates may not be recorded. More than 60 species of venomous snakes were identified in India. However, the ‘Big Four’ snakes including *Daboia russelii* (Russell's viper), *Naja* (Indian cobra), *Bungarus caeruleus* (common krait), and *Echis carinatus* (saw-scaled viper) are responsible for the majority of incidents and resulting deaths, disabilities and socioeconomic ramifications (Vaiyapuri et al., 2013; Chakma et al., 2020; Samuel et al., 2020). The state governments in India provide free healthcare for people through diverse settings ranging from primary health centres (PHC), community health centres (CHC), sub-divisional hospitals (SDH) and rural hospitals (RH) to tertiary care and medical college hospitals at the district level. Village-level support from accredited social health activists (ASHA - community health workers) may generally be absent in most cases as SBE is not included in the list of incentive activities for ASHAs. The state governments provide antivenom free of charge in tertiary care and other hospitals with facilities to administer intravenously. SBE treatment is also available in selective PHCs, SDHs and CHCs. However, due to the unstable infrastructure and insufficient number of staff, these healthcare facilities often prefer to refer patients to tertiary care hospitals. In addition, private healthcare is widely available across the country where people pay for their treatments. There are several ambulance providers in India including the service provided by the government, that respond to SBE emergencies via toll-free helpline numbers (specifically 108) (Gimkala et al., 2016). Access to ambulances and their response times may vary among states due to several factors such as the number of ambulances available, geographical terrain, time to reach the location, and distance of the location. However, a previous study reported that around 28,000 SBE victims in 2014 were transported to hospitals within 1 h of response time (from the time of the call to hospital admission) by emergency ambulance services which were available through the toll-free number 108 in 10 Indian states and 2 union territories (Gimkala et al., 2016). Despite this, a large proportion of victims may choose to use their own transportation due to a lack of awareness, the reliability of ambulance services and delayed arrivals of ambulance vehicles. India has a polyvalent antivenom produced against the ‘Big Four’ snakes and it is used for all envenomation cases. There are currently seven antivenom producers in India and the cost of antivenom is controlled by the Government of India (Whitaker et al. 2012). Antivenom is listed as an essential drug in all states and union territories except Ladakh. Health is considered a state matter in India, and therefore, the compensation for deaths and disability, claiming processes and the departments that manage them may vary in every state. The government's standard guidelines and protocols are available to support the clinical management of SBE in the country (National Health Mission, 2016). Moreover, some state governments provide insurance coverage for SBE treatments [e.g., the Tamil Nadu government provides the Chief Minister's Comprehensive Health Insurance Scheme (Government of Tamil Nadu, 2009)]. However, to benefit from such insurance, patients may need a bank account and seek treatment in a healthcare centre that is approved to use such a health insurance scheme. Several private hospitals either may not be approved or willing to get approval for government insurance policy schemes. Patients seeking treatment in such facilities must pay for the treatment.

Brazil is the fifth largest country in the world with an estimated population of over 215 million people (Government of Brazil, 2022). The country is made up of 26 states and one federal district (Government of Brazil, 2022). In Brazil, SBE is a major public health issue, and it affects numerous rural communities. An estimated 30,000 SBE incidents occur every year in this country resulting in approximately 4000 deaths (Schneider et al., 2021). Most SBE incidents are caused by snakes of the *Crotalus*, *Lachesis*, *Micrurus*, and *Bothrops* genera although *Bothrops* species (lanceheads) cause more incidents than the others since they are found throughout Brazil (Silva et al., 2019). The Ministry of Health in Brazil is responsible for managing SBE in the country, including the distribution of antivenom to main hospitals free of charge. Private hospitals may not be able to buy antivenoms for SBE treatment. However, the limited supply of antivenom may result in difficulties in accessing it by populations living in remote areas (e.g., tribal communities living in the Amazon Forest). Hence, the treatment may not reach all SBE victims when necessary (Cristino et al., 2021). Moreover, Brazil has other issues such as its biome diversity, the richness of snake species, and the gaps in socioeconomic indicators across areas, making it difficult to control and map the SBE issues. Thus, developing community health education programs in vulnerable regions and medical care settings for prevention, first-aid, and training has been demonstrated to be a significant strategy in mitigating SBE. The North region of Brazil encounters the most SBE incidents due to logistical, cultural, and financial difficulties as well as limited access to antivenom. For example, nearly one-third of total SBE incidents in Brazil were reported in the North region including Roraima and Amazonas states (Schneider et al., 2021). Data shows an uneven distribution of SBE issues within the country. Roraima is Brazil's northernmost state, presenting typical vegetation of the Amazon rainforest, the tropical climatic conditions observed here provide suitable habitat for a diversity of snakes throughout its territory. The stable annual temperatures (average of 27 °C) and high humidity provide the perfect conditions for reptiles to reproduce and thrive. Hence, Roraima is the state with the highest SBE incidence in Brazil, accounting for around 70 incidents per 100,000 people in contrast to other states (e.g., Amazonas state accounts for 50 SBE incidents per 100,000 people) (Alcântara et al., 2018). However, the actual number of SBE incidents in Roraima and Amazonas is likely to be much higher. Although underreported SBE incidents are a significant issue in all Brazilian territories, Roraima can be highlighted due to the scarcity of research investment, a high number of indigenous communities (more than 40% of the state), and the migration of people from Venezuela, since Roraima is the main gate to Brazil from Venezuela. In addition, the state has the least developed economy in the country and a fragile health system, lacking doctors and medical supplies in multiple regions. The State of Amazonas has the second-highest incidence of SBE cases within the Northern region (Schneider et al., 2021). It is noteworthy that the incidence of SBE has been constantly increasing since 2000, this may be due to deforestation, which makes the population more vulnerable. The loss of habitat resulting from deforestation displaces snakes and forces them into new habitats and into conflict with humans, increasing the prevalence of *Bothrops* species within habituated areas, these species are responsible for the most SBE cases (Alcântara et al., 2018; Beck et al., 2022). In Amazonas states, the estimated annual costs associated with SBE are over $6 million (USD) dollars, of which nearly $3 million is due to loss of productivity because of premature death and $1.5 million is due to lost productivity resulting from permanent disabilities (Magalhães et al., 2020).

## 4 A range of approaches used to educate rural communities

Here, we describe the community health education programs that have been developed and successfully implemented by various organisations in India and Brazil to promote awareness about snakebites. The details of the different approaches used, the level of outreach, and their impacts are documented.

**Fig. 1:**
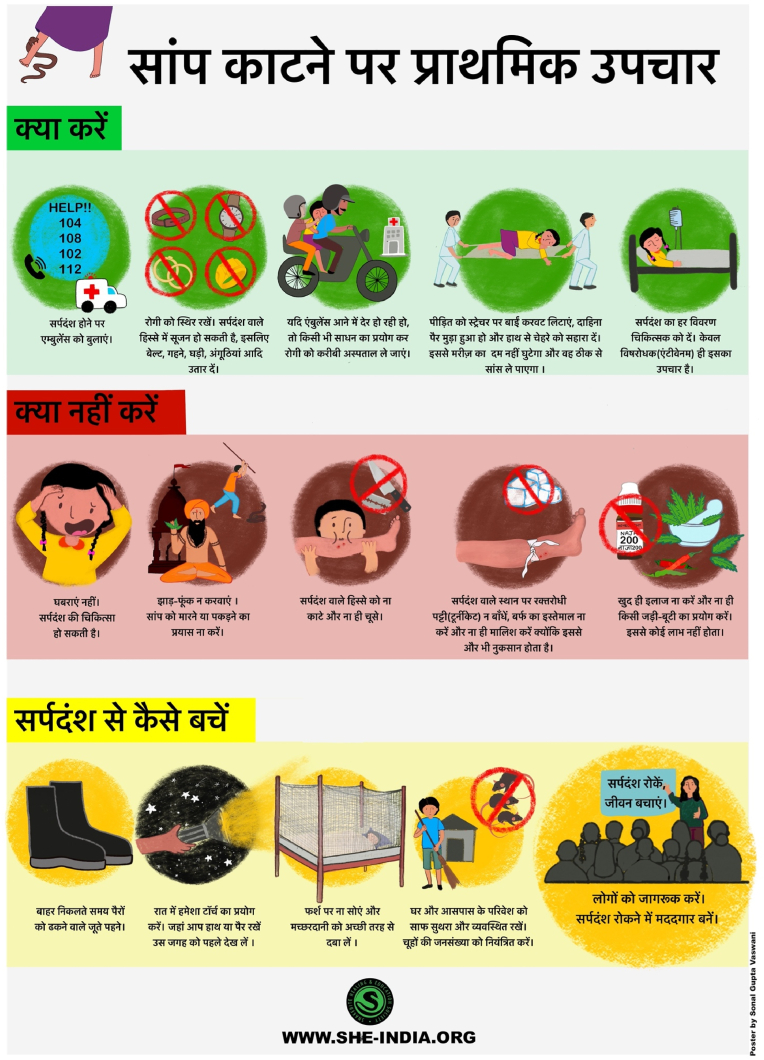
Snakebite information poster in Hindi developed by the SHE-INDIA organisation.

Snakebite Healing and Education Society (SHE-INDIA) (www.she-india.org) in India has been performing a wide range of community awareness activities to improve basic knowledge and treatment-seeking behaviour of rural communities for SBE. SHE-INDIA developed community awareness tools in line with the needs of the target audience. Their short videos of one to 6 min with essential information about SBE and its effects have been highly engaging and impactful in communities (SHE, 2020). Their videos were translated into multiple regional languages, which is an important element in India as it contains several states with different languages, traditions, and cultures. Notably, SHE-INDIA produced an advocacy film, ‘The Dead Don't Talk’ (SHE-INDIA 2018; SHE-INDIA, 2022a) which became a powerful advocacy tool for SBE in India. The film incorporated interviews with SBE victims and their families to portray their experiences following the incidents. The stories about SBE victims whose lives were dramatically changed due to SBE effectively demonstrated the necessity to seek immediate medical treatment and highlighted its resulting socioeconomic burden. SHE-INDIA has also produced posters (Fig. 1) and short videos to educate the communities about the prevention of SBE and appropriate first aid in 12 regional languages. The content is freely available on their website and social media pages. SBE awareness information and mitigation strategies are shared on their Facebook page (SHE-INDIA, SHE-INDIA), LinkedIn and Twitter. Similarly, SHE-INDIA has been involved in programs broadcasted by All India Radio (AIR 2022), podcasts, Tedx Talks, National level panel discussions (Council 2022) and a rural media platform (Gaon Connection TV, 2022).

In addition, SHE-INDIA developed Venom Response Teams comprising local animal rescuers, community educators and self-help groups in rural areas to support SBE victims and their families immediately after the incidents to ensure they seek prompt medical treatment. In collaboration with corporate companies and educational institutions, SHE-INDIA organised several film screenings, workshops, and seminars where SBE information has been disseminated as well as exchanging ideas from various groups and countries to learn successful approaches used in different regions. In several regions, animal rescue groups and local NGOs that work with the forest departments were utilised to disseminate key information about SBE. Their tools also covered instructions to stabilise SBE victims following bites and ensure immediate healthcare has been received. Since usage of the internet and social media is prominent throughout India, SHE-INDIA has utilised various social media platforms to exchange information about SBE and engage with the victims' families and local communities to spread science-based awareness information and ideas to reduce human-animal conflicts to further prevent SBE. This has been an impactful and accessible method utilised in their awareness campaigns. SHE-INDIA collaborates with several corporate companies to fund their projects with the strict understanding that the collaboration is a charitable activity and that the programs will not promote the company's products. The organisation has worked in 13 states of India and collaborated with the state-level health and forest departments to work on SBE prevention, control and capacity building of Primary Health centres and Community Health Centre doctors and nurses. The CMEs conducted by SHE-INDIA use theory and practical training through simulation models to teach the use of a suction catheter, bag valve mask and laryngeal mask airways to nurses and tracheal intubation to PHC and CHC doctors. SHE-INDIA has also created a WhatsApp group with clinicians, herpetologists, teachers, and social activists from different states to guide and support rural healthcare professionals in the management of SBE.

**Fig. 2:**
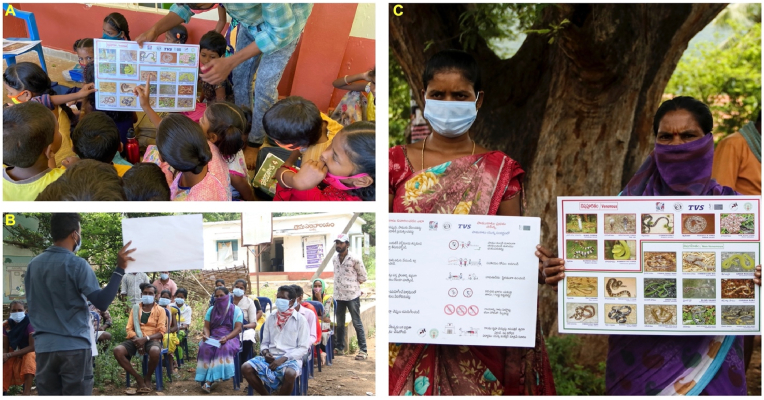
Snakebite Educational Programs from the Madras Crocodile Bank Trust. The campaign team has engaged with school children (**A**) and villagers (**B**) using information posters prepared in local languages (**C**).

The Madras Crocodile Bank Trust (MCBT) (https://madrascrocodilebank.org) is another organisation that has conducted valuable community-based studies to assess and improve the knowledge of SBE among rural communities. MCBT developed street plays and games for children promoting interactive learning about snakes and their safety measures. MCBT's Snakebite Mitigation Project conducts SBE awareness programs in 11 Indian states and has reached an estimated population of over 1.6 million through online activities and in-person assemblies in various locations. The videos (Sargunaraj, 2019) produced by MCBT were widely distributed and played a key role in several education programs. MCBT distributed educational handouts and installed over 400 A1-size posters with SBE information in various schools across India (Fig. 2). As a part of the project, walls of several schools and public areas were painted with SBE prevention and first aid information. MCBT is constantly developing efficient and sustainable educational models/tools to improve SBE awareness among rural communities. MCBT recently conducted a study to assess the communities outlook on protective equipment such as gumboots, torchlights, and mosquito nets and its ability to prevent SBE (Malhotra et al., 2021). The study highlighted the benefits and barriers of using the equipment which can be used to modify future programmes to ensure greater success. MCBT also conducts specialised training and capacity-building programs for the staff of the Forest and Fire Departments as well as snake rescuers across India.

**Fig. 3:**
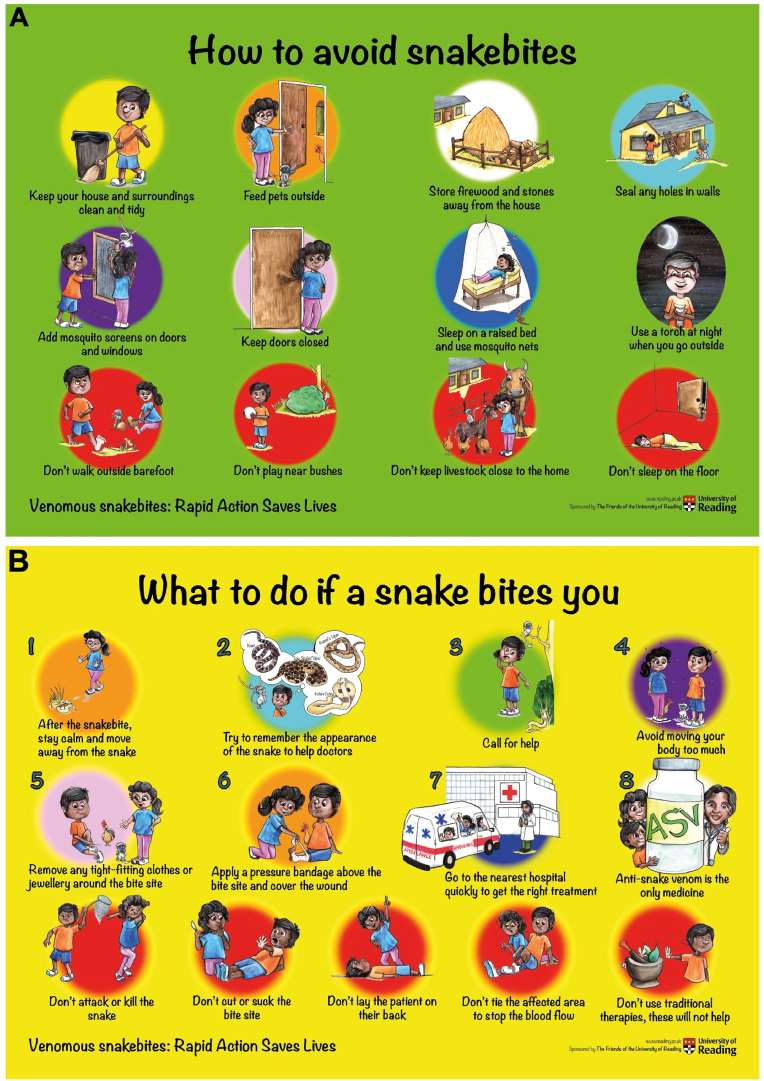
Highly engaging children-friendly SBE information poster prepared by the University of Reading. The Venomous Snakebites: Rapid Action Saves Lives team at the University of Reading has developed highly engaging posters on how to avoid snakebites (**A**) and what to do if bitten by snakes (**B**) using inclusive cartoon images that represent diverse ethnicities.

The University of Reading (UoR) (www.reading.ac.uk) in the United Kingdom has developed a multifaceted community health education program, namely ‘Venomous Snakebites: Rapid Action Saves Lives’ to specifically improve SBE awareness, education and clinical management in Tamil Nadu, a large state in Southern India (Samuel et al., 2020). In collaboration with various stakeholders including clinicians, conservationists, journalists, NGOs, local government officials and educational organisations, the UoR team have made remarkable advances in improving the knowledge surrounding SBE and the treatment-seeking behaviour of victims and their families. Through direct assemblies, we reached over 50,000 students in schools, colleges, and universities. Similarly, through direct engagement activities in rural villages, we reached over 350,000 people throughout Tamil Nadu. Using lectures, video documentaries, information leaflets, pocket guides and posters, the UoR team has increased the SBE knowledge following their campaign activities compared to the level of knowledge that people had prior to participating in said activities. The unique SBE information leaflets that they recently developed using cartoons that are inclusive and representative of a range of ethnicities helping to engage with the target population (Fig. 3) are critical for SBE awareness activities. We also made animation videos using these cartoons to engage with targeted communities (University of Reading, 2022a &b). Participants knowledge retention was analysed, and significant information retention was documented even 12 months after participation in campaign activities. In addition to direct engagement, the team has used mass media in the state and country to publish numerous articles with essential information about SBE prevention and first aid. The combined readership of only the top few media providers suggests that the key messages would have reached over 70 million people within the country. Similarly, short, and long video documentaries were broadcasted through various television channels and podcasts via radio. One of these video documentaries reached over 4 million people during its broadcast over two weekends during peak hours. Notably, they developed a dedicated Facebook page (Vaiyapuri, 2018) to disseminate their materials, and events and engage with members of communities to answer their queries relating to SBE. This platform has also enabled them to disseminate short video documentaries to the specifically targeted rural population of Tamil Nadu. They also engaged several other social media-based platforms to disseminate key information about SBE. To ensure the continuous education, dissemination of information and monitoring of impact, they recruited over 120 snake rescuers and volunteers as ‘local champions’ in rural villages, providing them with an adequate supply of materials. Recently, the UoR team engaged with school students and villagers in remote trial regions providing them with torches, school bags and geometry boxes with information about SBE. These materials were gratefully received and were critical in ensuring engagement with these populations. They were not only useful to spread the message, but also beneficial in promoting the adoption of appropriate preventive and first aid measures when bitten by snakes. This approach has substantially increased their reach and impact in improving SBE awareness and changing treatment-seeking behaviour. In summary, the UoR activities have led to the engagement of a substantial number of people within Tamil Nadu, and more widely in India within a short time through effective multifaceted community education approaches.

**Fig. 4:**
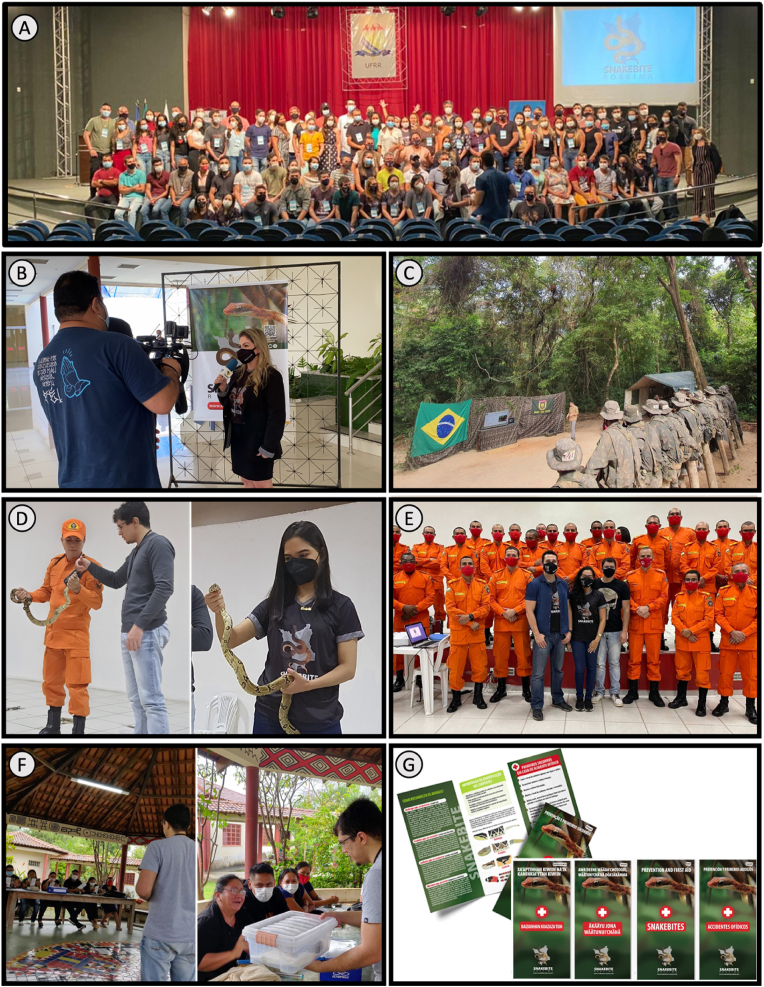
Snakebite Roraima Educational Program. (**A**) Snakebite training exclusively for Amazon Health Professionals was performed by Snakebite Roraima and FMT-HVD (2021). (**B**) Prevention campaigns disseminated through mass media. (**C**) Snakebite management training for Roraima's army (2022). (**D**) A fireman and a student learning how to handle a snake. (**E**) Snakebite training was performed for firefighters (2022). (**F**) Snakebite prevention campaign performed in an indigenous community (2022). (**G**) Snakebite Roraima illustrated, and printed material in different languages, including indigenous.

A few community education strategies have been developed in the northern region of Brazil and they were explained in detail in a previous study (Beck et al., 2022). In addition, the community education program coordinated by the Snakebite Roraima Research Group (www.snakebiteroraima.com) has made significant efforts in the state of Roraima and all over Brazil. The group covers a ‘Snakebite Prevention and Control Program’ (SPCP) to provide basic information on SBE preventative measures in different Roraima communities: rural populations, indigenous people, military personnel (army), and Venezuelan migrants (Fig. 4). In addition, the current activity of the group focuses on disseminating SBE knowledge throughout Brazil using their exclusive social media channel. The Snakebite Roraima group allows healthcare providers and students to benefit from their experience and improve their knowledge regarding the clinical management of SBE and prevention strategies. The Snakebite Roraima group indicators report that more than 20,000 people benefited from their program within different regions.

## 5 Methods to improve the skills and knowledge of healthcare professionals

SHE-INDIA organisation has been working with healthcare professionals in India to improve SBE treatment and outcomes. To build the nationwide SBE treatment capacity, SHE-INDIA recruited experts who routinely treat SBE to train numerous doctors, nursing staff and other rural healthcare workers in PHCs, sub-district hospitals, CHCs, and rural and district hospitals, improving their confidence in handling SBE victims in rural settings. SHE-INDIA organisation has also produced awareness films covering the importance of stabilising patients and transporting them to the local primary health centres, helping rural healthcare professionals in providing initial first aid and appropriate support for the victims before referring them for further treatment in tertiary care settings (SHE-INDIA 2021a; SHE-INDIA 2021b). This training ensured that referrals to tertiary care hospitals was reserved for critical cases.

The Venomous Snakebites: Rapid Action Saves Lives campaign has organised symposiums on clinical management of SBE to improve the skills and knowledge of rural healthcare professionals. These events attracted numerous clinicians, nurses, pharmacists, specialists, and medical officers. They covered a range of topics and medical aspects such as first aid, surgical procedures, kidney damage and issues with non-venomous snakebites for the clinical management of SBE. They also discussed the current issues with the standard protocols and guidelines provided by various healthcare authorities. These events enabled campaigners to create a network of experts which act as a forum to facilitate further discussion and support in handling SBE victims, their common symptoms, and rare complications. Notably, this network of healthcare professionals forged collaborative clinical research on various aspects of SBE using samples obtained from victims.

In Brazil, the Snakebite Roraima educational program is making huge efforts to improve knowledge about SBE at the clinical level, ensuring the adequate use of antivenoms through a Snakebite Training Program. The training programmes take undergraduate students from medical and nursing schools, physicians, nurses, and other healthcare staff (e.g., pharmacists, physiotherapists, and healthcare agents) from hospitals and basic healthcare units of the state and train them on the use of antivenoms and proper management of SBE victims. The existing guidelines from the Ministry of Health in Brazil have general recommendations for the clinical management of SBE, but it does not address pre-hospital care, first aid, storage, preparation or administration of antivenoms, wound care, auxiliary treatment for different local and systemic manifestations, referral to more complex health services, discharge criteria, determination of clotting time or the need to report cases to epidemiological surveillance systems. Hence, an integrated project in Brazil brought together a group of specialists (from the Dr Heitor Vieira Dourado Tropical Medicine Foundation (FMT-HVD), a tertiary care hospital for SBE in the Brazilian Amazon, and the Butantan Institute, the largest producer of antivenoms in the country to create SBE management guidelines and validate its contents. Their collective efforts resulted in new guidelines covering all these aspects to facilitate the clinical management of SBE including in rural settings (Rocha et al., 2022).

## 6 Engagements with local authorities to improve policies for SBE

Effective strategies to work with various stakeholders from diverse backgrounds within the government and NGOs to improve guidance and support for SBE awareness and prevention through education and learning are key to achieving a reduction in SBE incidences and resulting deaths. To ensure SBE is included in the existing national health programs in different countries, intervention is necessary both at the policy and field levels (Chakma et al., 2020). Hence, advocacy is critical to highlight the burden of SBE and the status of the vulnerable/affected population (livemint, 2022).

In India, the SHE-INDIA organisation collaborates with various stakeholders at central and state-level health and forest departments. They identified partnering with health and forest departments at the state and district level as the most successful model to be implemented at the grassroots level through their activities. At the district level, stakeholders including the panchayat leaders, the ASHA network and the Anganwadi workers (rural childcare centres) are important avenues through which community awareness messages can be delivered as these stakeholders work directly with the families at the village level and communities trust them. SHE-INDIA, therefore, engages the target population through local stakeholders to reduce the SBE burden. Moreover, they worked with district-level health teams to conduct CME programs for SBE based on the principles of emergency medicine that provide simulation-based practical training on performing intubation, the use of laryngoscope and suction tube, and the use of face masks and Ambu bags to provide ventilatory support and stabilise SBE patients.

The MCBT is working with government and non-government stakeholders to improve policies for SBE in India. MCBT has advised the Indian Council for Medical Research on publishing a white paper on SBE. Notably, MCBT is the NGO representative to the National Consultation on Prevention and Control of Snakebites by the Ministry of Health and Family Welfare, Government of India. The consultation has concluded to implement dedicative and monitoring strategies to mitigate SBE across the country. These strategies will be implemented across several state National Health Missions. MCBT produced a policy film to highlight the significance of SBE in northeast India. The film was shot in this region to realistically demonstrate the extent of the problem. This film shed light on the challenges with accessing good healthcare provision. MCBT and SHE-INDIA are regularly advocating for SBE to be declared a ‘Notifiable Disease’ in India under the Epidemic Diseases Act of 1987 (Government of India, 1987) as then it can receive much more attention from the government.

The team led by the UoR has engaged with the government health and other relevant authorities in Tamil Nadu at various levels. Their vision is to improve awareness, education, clinical management, and affordable care for SBE within this state. The UoR team organised conferences with relevant stakeholders to discuss various aspects of SBE, venom toxicology and developed strategic maps to achieve their vision. Their engagement with healthcare professionals and experts who are involved in medical education identified gaps in currently used curricula for medical and allied healthcare courses relating to SBE education, skills development, and training. As a result, they are in the process of developing curricula and training programs to improve education and training for students who are studying medical and allied healthcare programs. Their further engagement with professionals in the fields of agriculture, law, homoeopathy, and Ayurveda identified various aspects that can be improved by introducing SBE education and awareness in these areas. Furthermore, in discussion with educationists who teach a wide range of life science courses, they identified the areas where venom toxinology along with SBE education can be introduced to reveal the therapeutic potential of molecules present in venoms while educating them about SBE. As their initial step, they are involved in introducing SBE education and awareness in secondary school curricula in discussion with the Ministry of Education at the Government of Tamil Nadu. Discussions with key stakeholders from the Ministry of Health at the Government of Tamil Nadu identified the issues around collecting accurate statistics, antivenom production, its availability within the state and the necessity for improved SBE training for rural healthcare professionals to provide prompt treatment for victims and make referrals only when necessary. To reduce the burden arising from treatment costs in private hospitals, the team has engaged with a leading healthcare insurance provider and identified cheap insurance policies that enable victims to seek prompt treatment in private healthcare settings without worrying about the treatment costs.

In the Amazon region, the education of professionals has been taking place through partnerships with municipalities in the state of Amazonas and other states in the northern region. Thus, with the construction and validation of the snakebite management guide, a work team was established between the FMT-HVD and Butantan Institute, with the support of the Brazilian Ministry of Health. Although many activities have been performed by the team of the FMT-HVD, the success of research with the Ministry of Health is still emerging. Limited research funding for neglected areas such as the Northern region, has resulted in poor investment into SBE policies in recent years. Although some public employees are dedicated to carry out campaigns and projects to mitigate SBE, funding is frequently denied making it impossible to carry out their actions.

## 7 Impact of community health education programs

Measuring the impact following public awareness activities is a challenging task especially in large countries. SHE-INDIA organisation provides their awareness materials in multiple languages free of charge through their online and social media platforms. The direct requests and number of views/downloads demonstrates the impact their tools have. There is also the free distribution and use of educational materials by groups that have procured the content through the NGOs working with SHE-INDIA. The impact in those cases can only be extrapolated by observing the health-seeking behaviour of the local populace following bites. The engagement with local villagers and students in educational organisations is demonstrated through the number of people who attended the events. In the last decade, SHE-INDIA has performed more than 1200 workshops. Through the Venom Response Team in local areas, SHE-INDIA envisages improving the recovery of SBE patients confirming that the recovery rate is higher than that before the development of such local teams.

The MCBT has conducted questionnaire-based surveys to assess the impact of their education programs. They conducted surveys pre- and post-education programs to understand the base knowledge of the people and to evaluate how much knowledge has been gained through involvement in activities. The surveys were conducted at different time intervals to get more diverse results. Based on these results, there has been a significant improvement in awareness in communities that attended the educational outreach programs. The survey had simple questions, translated into regional languages to get more accurate responses from beneficiaries. Audio and video testimonials were also recorded to assess impact. In online platforms, MCBT's work had reached over 1 million views. MCBT's work was recognised by several regional governing bodies and taken as a model to upscale.

The UoR has utilised robust methods to measure the impact of its activities in Tamil Nadu. For example, to measure the impact of SBE awareness among students and villagers, they employed a pictorial questionnaire with specific tasks on SBE prevention and first aid (Samuel et al., 2020). This questionnaire was used before their activities to capture their prior knowledge of SBE, immediately after their activities to determine their knowledge change, and after 12 months to analyse their long-term retention of knowledge. This method demonstrated the impact of their activities in significantly increasing the knowledge of villagers and students immediately after their activities, and more than 85% of people retained the knowledge 12 months later. To analyse the treatment-seeking behaviour of SBE victims and their relatives, they analysed data from a snakebite referral hospital before and after their activities. Remarkably, following their activities, a majority of SBE victims arrived at the hospital within 4 h following the bites and without practising any inappropriate first aid. This earlier arrival in the hospital resulted in early discharge and reduced treatment costs. This data demonstrated the translation of knowledge into treatment-seeking behaviour of villagers following SBE. The knowledge, skills and training they provided resulted in a change in clinical practice at various places as demonstrated by the number of questions that they received through their network forums and personal emails sent to the team. Following the discussion with the Ministry of Health, the Government has increased the family's annual income level to be eligible for the Tamil Nadu Chief Minister's Comprehensive Insurance Scheme for people who earn a specific amount of money every year. The insurance provides treatment coverage for SBE along with a range of other illnesses in private hospitals in Tamil Nadu.

In the Amazon region, each municipality often has only one hospital at its headquarters, where SBE victims should travel to receive antivenom treatment. There is no antivenom available in rural health facilities where most incidents occur. Thus, access to antivenom is very limited due to long distances, and travel to the nearest hospital can even take days. Instead of being distributed to rural and indigenous health facilities, where most basic health problems are resolved by nurses, antivenom treatment is generally available only at the municipal headquarters, where physicians are present. Therefore, the decentralisation of antivenom treatment, which aims to promote the immediate and effective action of health professionals in the place where it occurs, will favour the prompt treatment and prevention of SBE-induced deaths and disabilities. This proposed action consists of care in health units under the supervision of non-medical professionals, when there is no doctor available, and this can be performed by a properly trained nurse. This is expected to increase the accessibility to antivenom treatment, reduce the time interval between the bite and treatment, and consequently, have a better prognosis for victims. As a first step in the decentralisation process, the specialist team guides the management of SBE and antivenom treatment using multidisciplinary care protocols. Moreover, there were a few campaigns relating to venomous animals led by the Ministry of Health through the *Programa Nacional de Controle do Ofidismo* (PNCO, National Program for the Control of Snakebites) in 1986. In 1989, this program was updated to the *Programa Nacional de Controle dos Acidentes por Animais Peçonhentos* (PNCAAP, National Program for the Control of Accidents by Venomous Animals). As a result of Snakebite Programs, the Ministry of Health recently launched the 5th edition of the *Guia de Vigilancia em Saúde* (GVS, Health Surveillance Guide), which elucidates strategies for surveillance, prevention and control of selected diseases important to public health, including SBE, leprosy, tuberculosis, and dengue (Malhotra et al., 2021). In parallel, researchers and professors residing in areas of the Brazilian Amazon Forest continue to perform SBE educational projects using their initiatives and funding. Although still modest, the number of SBE incidents in Brazil has begun to decrease in the last 3 years because of their educational projects.

## 8 Limitations

The approaches and resources described in this article were successfully developed and used in India and Brazil. Although these methods along with resources can be easily translated to any other countries or specific communities, modifications may be necessary to meet the needs of target populations. Moreover, such interventions must involve local people or groups who are trusted by rural communities as without their support, it may be difficult to engage and impact the population with awareness activities. Therefore, it is important for experts who can provide proper scientific, clinical, and epidemiological advice to engage with local people, volunteers or NGOs who are interested in these activities to design and develop people-centred approaches to improve the impact. Such methods are more likely to impact behavioural changes among rural people to reduce SBE and seek prompt medical treatments following bites. In addition, SBE researchers and clinicians should understand that making policy changes to include SBE in the existing health programs and impact on societies may take a long time due to the complexities associated with healthcare systems and the priorities of the governments during annual budgetary allocation for health risks in rural populations. Researchers must carefully design the impact measurement methods and tools at an earlier stage when they decide on the activities and target populations. It is critical to demonstrate the impact of any activities with rural communities to pave the way for future events and changes. The researchers should also be aware of ethical considerations, the necessity to obtain consent from target people or patients, when necessary, seeking appropriate permissions from relevant authorities, copyright issues when using licensed materials and risks in using media and social media. The funding for such awareness activities should also be carefully considered during the design of the project as abandoning the activities without completion is likely to cause reputational damage to the institutions and researchers, which may affect their future engagement with those communities.

## 9 A brief summary of similar snakebite health education activities in other countries

While much focus of this paper has been on the efforts to tackle SBE in India and Brazil, there are many other non-profit organisations, initiatives and charities that help to tackle the SBE problem worldwide. Asia, south America, and Africa are the major three hotspots for SBE, due to their abundance of venomous snakes’ life, poor infrastructure and healthcare provision associated with their low socioeconomic status. In these parts of the world a large majority of people live and work in rural areas, in agriculture and in close proximity to snakes. It has been estimated that 1 million snakebites occur every year in sub-Saharan Africa, with 100,000–500,000 envenomations and 10,000–30,000 deaths (Chippaux, 2011). This number is greatly underreported for the same reasons described above in India and Brazil. Antivenom use within sub-Saharan countries depends on its accessibility including financial burden. In some parts of sub-Saharan Africa such as the Democratic Republic of Congo, antivenom is not available, in others, it is not available in remote settings where most bites occur and many antivenoms are unsuitable as they have been raised against snakes that are not from Africa or have been poorly purified (Chippaux, 2011; Warrell, 2008). The Health Action International (HAI) is an independent non-profit organisation that advocates access to effective quality and affordable medicines for all. Their HAI snakebite project works in collaboration with the Global Snakebite Initiative in sub-Saharan Africa, specifically Uganda, Zambia and Kenya to help mitigate SBE. Their project focusses on gathering data surrounding SBE, healthcare provision and antivenom treatment to gain a better understanding of the true burden faced by people in these countries. They have created civil society driven multi-stakeholder groups of snakebite experts that utilise the data collected to call for changes in policy. Education outreach projects provide local communities with the tools and resources needed to learn how to minimise conflict with snakes and reduce SBE, meanwhile teaching them how to treat SBE victims and what to do in the case of a snake bite. Their goals are to empower the people through awareness and education, make SBE reporting mandatory, ensure antivenom is of high quality, financially affordable and available to all. As with most initiatives, they have identified the importance of educating health care workers about SBE and how to properly care for their patients. This care extends beyond the primary health care provision and into the rehabilitation of patients that develop long-term morbidities following SBE, helping to improve their quality of life (HAI, 2022). In addition, another team of researchers introduced the use of a ‘snake song’ that was produced in Tamil (as well as in Zulu) to engage with children and educate them about what to look for and why should they care about SBE in a musically engaging manner (Erickson et al., 2020).

## 10 Future directions

As highlighted in the WHO's strategic roadmap, empowering and engaging the communities and collaborating with Government and non-government stakeholders is a critical approach to mitigating SBE incidents and reducing the deaths, disabilities, and socioeconomic impacts on victims and their families. In recent years, several researchers have been involved in public engagement activities with rural communities in certain countries. However, more activities and a greater range of professionals and organisations including faith healers, alternative medicine practitioners, religious leaders, educationists, students, local champions, NGOs, hospitals, health workers and community centres should be involved and motivated to disseminate SBE awareness and mitigate the disease burden. Notably, various funding agencies across the world should understand the importance of multifaceted public engagement activities to mitigate SBE and save lives and consider funding such activities. It has been demonstrated that these activities are relatively cheap, but are powerful tools to mitigate SBE incidents, deaths, and disabilities. Involving vulnerable communities and understanding their needs will also improve research success in developing better diagnostic and therapeutic approaches for SBE. Overall, we conclude that multifaceted community health education programs in collaboration with numerous stakeholders will create a measurable impact and reveal the complexities/issues that need to be addressed in those communities.

## Ethical statement

This research was conducted according to the Declaration of Helsinki and the ethical guidelines of the relevant institutions. The awareness activities and design and distribution of materials do not have any ethical concerns. However, the data collected from people and patients were covered under institutional ethical approvals as necessary.

## Credit author statement

Sakthivel Vaiyapuri: Study design, Formal analysis, Validation, Investigation, Resources, Data curation, Writing – original draft, Writing – review & editing, Visualization, Supervision, Priyanka Kadam: Study design, Formal analysis, Validation, Investigation, Resources, Data curation, Writing – original draft, Visualization, Supervision, Gnaneswar Chandrasekharuni: Study design, Formal analysis, Validation, Investigation, Resources, Data curation, Writing – original draft, Visualization, Supervision, Isadora S. Oliveira: Resources, Data curation, Writing – original draft, Visualization, Subramanian Senthilkumaran: Validation, Investigation, Resources, Data curation, Anika Salim: Study design, Formal analysis, Validation, Investigation, Resources, Ketan Patel: Formal analysis, Resources, Writing – review & editing, Jacqueline de Almeida Gonçalves Sachett: Study design, Formal analysis, Resources, Writing – review & editing, Manuela B. Pucca: Study design, Formal analysis, Validation, Investigation, Resources, Data curation, Writing – original draft, Visualization, Supervision.

## Declaration of competing interest

The authors declare that they have no known competing financial interests or personal relationships that could have appeared to influence the work reported in this paper.

## Data Availability

No data was used for the research described in the article.
